# SOCIOECONOMIC AND GENETIC DETERMINANTS OF COGNITIVE FUNCTION IN THE AGING POPULATION IN KAZAKHSTAN

**DOI:** 10.1093/geroni/igae098.3220

**Published:** 2024-12-31

**Authors:** Adil Supiyev, Angela Gutierrez, Graciela Muniz Terrera

**Affiliations:** Ohio University Heritage College of Osteopathic Medicine, Athens, Ohio, United States; Western University of Health Sciences, Pomona, California, United States; Ohio University, Athens, Ohio, United States

## Abstract

Objective There is limited evidence about cognitive performance of older adults in Kazakhstan, a Central Asian nation that was formerly part of the Soviet Union. Our study aims to fill the gaps by assessing cognitive function and its determinants in the population of Astana city and its surrounding rural areas. Methods Between 2012 and 2015, 954 older adults aged 50–74 from urban and rural areas in Central Kazakhstan participated in this study (response rate: 59%). Trained practitioners administered four neuropsychological tests to assess cognitive function, including verbal memory, verbal fluency, attention, and concentration. Additionally, a standardised protocol was used, consisting of a structured questionnaire, objective physical examination, and blood sample collection. Results After controlling for age, sex, and cardiometabolic risk factors, our analysis revealed significant associations between cognitive function and residence, ethnicity, education levels, and selected socioeconomic factors. Notably, APOE4 status showed a strong association with cognitive decline, consistent with prior research. Furthermore, individuals with higher deprivation levels, men, rural residents, and those with lower education levels demonstrated significantly poorer cognitive function outcomes. Conclusion Understanding the association between cognitive function and selected socio-demographic factors within the Central Asian region contributes significantly to global health insights. The contrasts observed in residential environments and levels of deprivation emphasize the urgency of targeted interventions aimed at mitigating cognitive decline risks among vulnerable populations worldwide.

